# Planar Micro-Supercapacitors with High Power Density Screen-Printed by Aqueous Graphene Conductive Ink

**DOI:** 10.3390/ma17164021

**Published:** 2024-08-13

**Authors:** Youchang Wang, Xiaojing Zhang, Yuwei Zhu, Xiaolu Li, Zhigang Shen

**Affiliations:** 1 Beijing Key Laboratory for Powder Technology Research and Development, Beihang University, Beijing 100191, China; by1805149@buaa.edu.cn (Y.W.); by2105121@buaa.edu.cn (Y.Z.); wyc_fendou@163.com (X.L.); shenzhg@buaa.edu (Z.S.); 2 School of Aeronautic Science and Engineering, Beihang University, Beijing 100191, China

**Keywords:** graphene, aqueous ink, rheological properties, micro-supercapacitors, high power density

## Abstract

Simple and scalable production of micro-supercapacitors (MSCs) is crucial to address the energy requirements of miniature electronics. Although significant advancements have been achieved in fabricating MSCs through solution-based printing techniques, the realization of high-performance MSCs remains a challenge. In this paper, graphene-based MSCs with a high power density were prepared through screen printing of aqueous conductive inks with appropriate rheological properties. High electrical conductivity (2.04 × 10^4^ S∙m^−1^) and low equivalent series resistance (46.7 Ω) benefiting from the dense conductive network consisting of the mesoporous structure formed by graphene with carbon black dispersed as linkers, as well as the narrow finger width and interspace (200 µm) originating from the excellent printability, prompted the fully printed MSCs to deliver high capacitance (9.15 mF∙cm^−2^), energy density (1.30 µWh∙cm^−2^) and ultrahigh power density (89.9 mW∙cm^−2^). Notably, the resulting MSCs can effectively operate at scan rates up to 200 V∙s^−1^, which surpasses conventional supercapacitors by two orders of magnitude. In addition, the MSCs demonstrate excellent cycling stability (91.6% capacity retention and ~100% Coulombic efficiency after 10,000 cycles) and extraordinary mechanical properties (92.2% capacity retention after 5000 bending cycles), indicating their broad application prospects in flexible wearable/portable electronic systems.

## 1. Introduction

The great progress of flexible portable/wearable electronic devices, such as micro-robots, implantable medical devices, and wearable health monitoring systems, etc., toward miniaturization, light weight, integration, and self-powering, has greatly increased the requirement for miniature energy storage systems, especially planar microbatteries (MBs) and micro-supercapacitors (MSCs) [1,2]. MSCs exhibit advantages over MBs with high rate capability, high safety, and long cycle life [3,4,5,6]. In addition, planar MSCs have stimulated considerable interest due to their distinctive interdigitated structure that enables extremely short ion diffusion paths, demonstrating their ability to be directly integrated with miniaturized electronics on the side of an insulated substrate as an independent micropower source or a miniature energy harvesting device [7,8,9]. Active materials, fabrication methods, and geometrical configurations of electrodes, are crucial for realizing the high-performance electrochemical performance of MSCs [10,11,12]. 

In terms of active materials, conducting polymers, transition metal oxides, and hydroxides exhibit high theoretical capacitance, but low electrical conductivity, a narrow operating window, and poor electrochemical reversibility limit their development [13]. Carbon materials, on the other hand, containing graphene, carbon nanotubes, graphite, activated carbon, and onion-like carbon, have received widespread attention due to their outstanding chemical stability and wide operating window. Among them, graphene is a desirable electrode material for MSCs due to its low toxicity, extraordinary electrical conductivity, high specific surface area, and excellent intrinsic double-layer capacitance (21 μF∙cm^−2^) or theoretical capacitance (550 F∙g^−1^) [10,14]. However, the simple and large-scale fabrication of graphene electrodes compatible with various flexible substrates remains a great challenge.

Significant advancement has been made in fabrication methods for graphene-based interdigitated electrodes, such as photolithography, laser scribing, electrophoretic deposition, and oxidative etching; however, the complexity of the processes, as well as the specific requirements for flexible substrates, limit their application for scalable preparation of MSCs [12,15,16,17]. Solution-based printing technology overcomes the limitations of these methods and becomes an alternative solution [11,18]. Screen printing provides a cost-effective strategy for integrating graphene into a robust and practical printing process that is compatible with a variety of substrates [19]. Moreover, screen printing demonstrates the highest deposition rates (weight of material deposited per unit time) and high throughput fabrication with a variety of complex customized patterns while allowing thicker films, thus increasing the area loading of graphene [20,21]. However, the use of high boiling points and toxic solvents such as N-methylpyrrolidone (NMP), N,N-dimethylformamide (DMF), acetone, and dimethylsulfoxide (DMSO) increases the post-processing time and hazards to humans, making the development of aqueous graphene inks a promising direction [22,23]. However, water as a solvent has several drawbacks, including: (1) rapid evaporation increases the viscosity of inks due to its low boiling point, (2) high surface tension promotes graphene agglomeration, and (3) the low viscosity of the aqueous binder leads to unsuitable ink rheology [24]. Alcohols have been introduced as co-solvents to solve these problems, but the influence of alcohols on the properties of aqueous graphene inks has rarely been investigated. On the other hand, the electrochemical performance of MSCs relies heavily on the geometrical configuration of the electrodes, with narrower finger width and interspace resulting in increased specific capacitance and power density [16,17,25]. Therefore, the requirement for high-resolution screen printing poses a challenge for high-concentration graphene inks with suitable rheological properties.

Herein, we fabricated aqueous graphene conductive inks based on a ternary hybrid solvent (water/ethanol/ethylene glycol), whose rheology was adjusted by varying the ratio of glycol and ethanol. The fully printed MSCs with narrower finger widths and interspaces exhibit a high area-specific capacitance of 9.15 mF∙cm^−2^, an area energy density of 1.30 µWh∙cm^−2^, and an area power density of 89.9 mW∙cm^−2^. Impressively, fully printed MSCs with such ultrahigh area power density outperform most carbon-based MSCs, providing ultrafast charging and discharging capabilities due to superior electrical conductivity and low ion diffusion impedance. In addition, our screen-printed MSCs demonstrate excellent mechanical flexibility, cycling stability, and compatibility with a variety of substrates, manifesting their great potential for future printed microelectronic devices.

## 2. Materials and Methods

### 2.1. Materials

Natural graphite flakes (≥99.8%, 325 mesh, NO. 43209) were purchased from Alfar Aesar (Shanghai, China). Carbon black (EC-600JD, DBP adsorption 4.8–5.1 mL∙g^−1^, surface area 1400 m^2^∙g^−1^) was purchased from Akzo Nobel (Shanghai, China). Waterborne polyurethane (F0402, 32 ± 5%) was obtained from Shenzhen Jitian Chemical Co., Ltd. (Shenzhen, China). Dispersant (Disper 850, 50%) was bought from Shenzhen Tongtai Chemical Technology Co., Ltd (Shenzhen, China) and defoamer (NXZ, 99%) was acquired from San Nopco (Shanghai, China). Polyvinyl alcohol (PVA1788, DH 87.0–89.0%) was obtained from Shanghai Macklin Biochemical Technology Co., Ltd (Shanghai, China). Deionized water (DI), ethanol (EtOH, ≥99.5%), ethylene glycol (EG, ≥99%), carboxymethyl cellulose (CMC) (Mw~250,000, DS = 0.7), and sulfuric acid (H_2_SO_4_, 98%) were bought from Beijing Ke’ao Bio-Tech. Co., Ltd. (Beijing, China).

### 2.2. Preparation of Aqueous Graphene Ink

**Figure 1:**
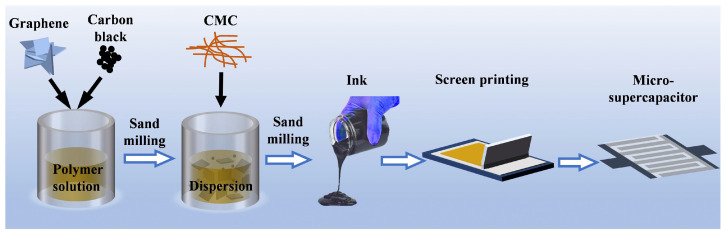
Schematic synthesis of aqueous graphene conductive ink and screen-printed preparation of MSCs.

The schematic synthesis of aqueous graphene conductive ink and screen printing for MSCs is shown in Figure 1. Briefly, the binder, i.e., aqueous polyurethane (F0402), and additives, including dispersant (Disper 850) and defoamer (NXZ), were dissolved in a solvent mixture of deionized water (DI)/ethanol (EtOH)/ethylene glycol (EG) to obtain a polymer solution. Then, graphene and carbon black were added to the polymer solution at a ratio of 85/15, and a homogeneous dispersion was obtained by mixing at a high speed of 2000 rpm for 8 h in a horizontal sand mill (NMM-1L, Boyee, Shenzhen, China). Finally, the thickener CMC (one of the additives) was added to the dispersion and continued to mill for 30 min, and a high concentration of graphene ink suitable for screen printing could be obtained. The mass fractions of solvent, binder (according to the active ingredient), conductive fillers, and additives in the ink were 77.6%, 4.8%, 12%, and 5.6%, respectively, of which the additives were composed of dispersant 3.6%, defoamer 0.5%, and CMC 1.5%. The solvent contained 50 wt% of deionized water and 50 wt% of alcohol, while the ratio of EG and EtOH was set to 0.25/0.67/1.5/4 for regulating the rheology of inks, and thus the corresponding inks were denoted as Ink–0.25, Ink–0.67, Ink–1.5, and Ink–4, respectively.

### 2.3. Screen Printing of Inks and Assembly of Micro-Supercapacitors

Screen printing was carried out using a screen printer (LTA 6080, LingTie, Xiamen, China) with the stencil placed about 5 mm above substrates, i.e., PET foil, PI foil, or paper, which were held against the base of the screen printer by vacuum suction. The inclined polyurethane squeegee formed an angle of 60° with the stencil and was printed at a speed of 50 mm∙s^−1^. Stencils with 325 meshes were used to print the fine lines for testing the resolution and interdigitated electrodes of MSCs. All wet printed patterns were dried at 80 °C for 15 min to remove the solvent. To investigate the effect of post-treatment on conductivity, as-dried printed patterns were compressed at 70 °C and 24 mm∙s^−1^. Flexible MSCs were assembled by drop-casting a hydrogel-polymer electrolyte (4 g of PVA1788 and 4 g of H_2_SO_4_ dissolved in 40 mL of DI under stirring at 90 °C until the solution became transparent) onto the projected area of as-dried interdigitated electrodes and resting for 24 h at room temperature.

### 2.4. Characterization

The morphology of materials was characterized by field emission scanning electron microscopy (FE-SEM) (10 kV, SIGMA, Zeiss, Oberkochen, Germany), transmission electron microscopy (TEM) (200 kV, JEM-2010F, JEOL, Tokyo, Japan), atomic force microscopy (AFM) (ScanAsyst Air mode, Bruker MultiMode 8, Bruker, Santa Clara, CA, USA), and optical microscopy (A2m, ZEISS, Oberkochen, Germany). The quality of graphene was examined using X-ray photoelectron spectroscopy (XPS) (1487.2 eV, ESCALAB 250Xi, Thermo Fisher Scientific, Waltham, MA, USA), and Raman spectroscopy (514 nm, Rm2000, Renishaw, Gloucestershire, England) to determine the type and level of defects. Steady state and dynamic rheological properties of inks were investigated at 25 °C using a MCR92 rheometer (Anton Parr, Graz, Austria) equipped with parallel plates of 50 mm diameter and 1 mm spacing. Before the tests, all the inks between parallel plates required standing for 5 min to eliminate pre-shearing, and the exposed edges of the inks were covered with a thin layer of silicone oil to prevent solvent evaporation. Steady-state flow step tests were performed at shear rates from 0.1 to 1000 s^−1^ to measure shear viscosity, and three-interval thixotropy tests (3ITT) were conducted by simulating the screen-printing process in three intervals (0.1 s^−1^ shear rate for 50 s, 100 s^−1^ shear rate for 40 s, 0.1 s^−1^ shear rate for 180 s) to investigate the thixotropic behavior of inks. Stress sweep tests in the range of 0.1–1000 Pa were carried out at 1 Hz in oscillatory mode. The data were analyzed using RheoCompass 1.24 software to obtain the energy storage modulus (G′), yield stress (τ*_y_*), and flow stress (τ*_f_*). τ*_y_* refers to the end of the linear viscoelastic range, while τ*_f_* represents the intersection of the G′ and G″ curves. The electrical conductivity of printed patterns was measured by a four-probe tester (KDY-1, Kunde, Guangzhou, China). All electrochemical measurements of MSCs were conducted at 25 °C using an electrochemical workstation (Zennium Pro, Zahner, Kronach-Zeilern, Germany). Cyclic voltammetry (CV) measurements were performed at a scan rate ranging from 1 to 200 V∙s^−1^. Galvanostatic charge/discharge (GCD) measurements were performed at different current densities ranging from 0.05 to 1 mA∙cm^−2^. Electrochemical impedance spectroscopy (EIS) tests were conducted in the frequency range of 0.01–10 kHz by applying an AC amplitude of 5 mV. Cycling capability experiments of MSCs were performed on a battery test system (CT2001A, LANHE, Wuhan, China). Bending tests of printed patterns and MSCs were carried out on a tensile testing machine (HSV, Handpi, Leqing, China). Detailed calculations about MSCs are available in the supporting information.

## 3. Results and Discussion

### 3.1. Morphology and Quality of Graphene

**Figure 2:**
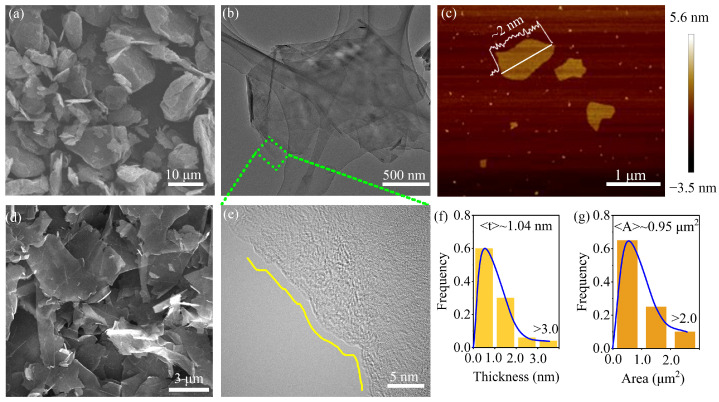
(**a**) SEM image of pristine graphite before exfoliation. (**b**) TEM image of several stacked graphene nanosheets. (**c**) A typical AFM image of graphene. (**d**) SEM image of graphene after exfoliation. (**e**) HRTEM image of the rectangle region in (**b**). Histograms of the (**f**) thickness distribution and (**g**) area distribution of graphene.

Graphene was prepared from the exfoliation of graphite by jet cavitation [26,27]. The dimensions and defects of graphene were initially investigated due to their significant influence on the rheology of inks and electrical conductivity, as well as the electrochemical properties of printed devices [28,29]. SEM was utilized to compare the morphology of pristine graphite before and after exfoliation. Natural graphite, characterized by a bulk structure with a lateral size of 10–20 μm and thickness in several micrometers (Figure 2a), was successfully exfoliated into amounts of graphene nanosheets with a lateral size of 1–5 μm (Figure 2d), which are stacks of fewer-layer graphene (FLG), as depicted in the transparent TEM image (Figure 2b). Impressively, the HRTEM image of the square region exhibits the characteristic of single-layer graphene, presenting only a stripe at the edge, as shown in Figure 2e. The morphology of exfoliated graphene was further examined using AFM. Figure 2c demonstrates a graphene nanosheet with a thickness of about 2 nm. Due to the potential presence of residual solvents and impurities between graphene and the substrate, the thickness of single-layer graphene observed in the AFM image may exceed 0.334 nm. Therefore, it can be concluded that graphene in Figure 2c consists of 1 to 3 layers. Figure 2f and g illustrate the histograms of area and thickness distribution obtained by counting AFM images of 113 graphene nanosheets. The average thickness and area of graphene nanosheets prepared by jet cavitation methods are 1.04 nm and 0.95 μm^2^, respectively. Additionally, approximately 60% of graphene is composed of 1–3 layers with a thickness of less than 1 nm (Figure 2f). More significantly, the lateral size of graphene exfoliated through jet cavitation exceeds that of graphene produced through the majority of other liquid-phase exfoliation techniques [26].

**Figure 3:**
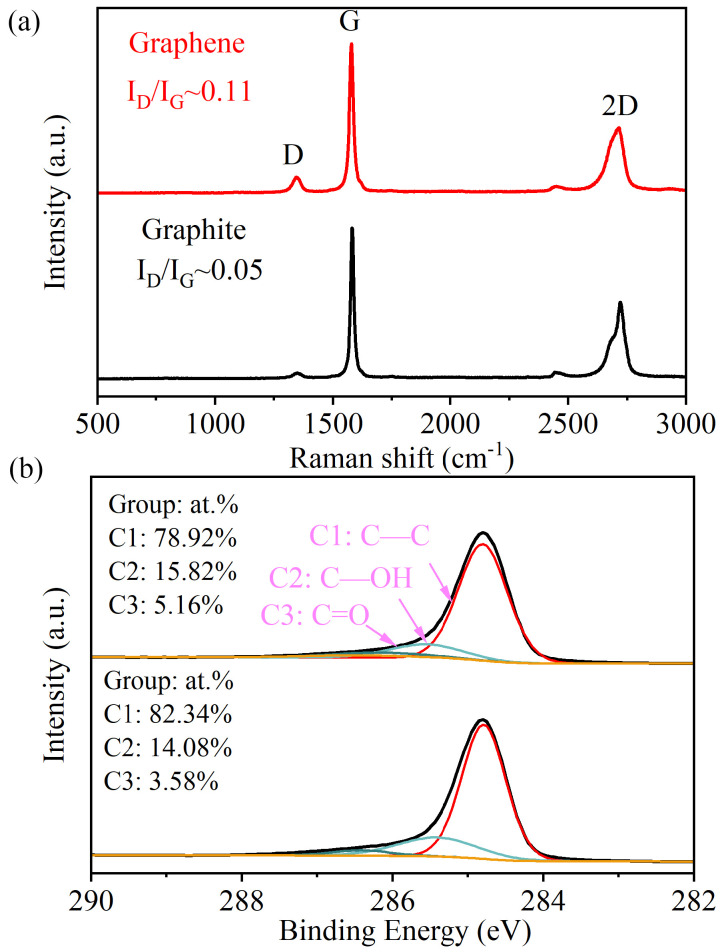
(**a**) Raman spectra and (**b**) C1s XPS of pristine graphite and graphene.

Raman spectroscopy and XPS were used to analyze the quality of graphene. As depicted in Figure 3a, the 2D band of graphene exhibits greater symmetry and lacks shoulder peaks in comparison to natural graphite, thus aligning with the characteristics previously reported for few-layer graphene. [30,31] The defect extent of graphene is determined by I_D_/I_G_, which represents the ratio of the intensity of the D band to the G band. In this work, the I_D_/I_G_ value of graphene (~0.11) is slightly higher than that of natural graphite (~0.05), but much lower than graphene oxide and reduced graphene oxide [32,33]. The D band, much stronger in the spectrum of graphene, can be attributed to edge defects and lattice disorder [34]. Furthermore, the unbroadened G band suggests that the basal plane structure of graphene remains unaffected [35]. Consequently, the elevated defect level of graphene originates primarily from edge defects. In fact, edge defects are inherent and unavoidable in graphene nanosheets due to the increase in total edge length per unit mass with an increase in the number of nanosheets (resulting in a decrease in lateral size) [36]. The carbon atoms at the edges undergo oxidation due to the presence of free radicals or ions that are generated by the decomposition of water at high temperatures and pressures. This reaction occurs in the small areas created by the bursting of cavitation bubbles [30,37]. As a result, C–OH and C=O bonds are formed (Figure 3b), leading to a slight increase in the oxygen content from 3.60% to 4.59% (Supplementary Material, Figure S1), which can increase the number of interaction sites of graphene with other compounds to improve its dispersion in water. Therefore, through the process of jet cavitation, bulk graphite can be effectively exfoliated into larger-size FLG with well-preserved basal planes, and it is anticipated that this distinctive structure will showcase superior electronic and electrochemical characteristics.

### 3.2. Rheological Behavior and Printing Performance of Graphene Inks

The performance of printed patterns is influenced by the behavior of screen printing, which is, in turn, determined by the rheological properties of the ink. Appropriate rheological properties result in the production of finer printed patterns with smoother edges, as well as facilitating the reduction in air bubbles and defects in the print patterns, resulting in enhanced electrical conductivity. Table S1 presents the physicochemical parameters of different materials, and it is evident that EtOH and EG have Hansen Solubility Parameter Distance (R_a_) values closer to graphene than water. Consequently, they can enhance the dispersion of graphene in water, as confirmed by other studies [38,39]. Furthermore, EtOH and EG can reduce the surface tension of the ink, thereby increasing its wettability on the substrate. Although EtOH, a low-boiling solvent, is more advantageous than ethylene glycol in improving the dispersion and wettability of aqueous graphene inks, its rapid volatilization results in an increase in the viscosity of the ink, which hampers the printing process and may even cause mesh blockage. Therefore, the presence of EG, which has a higher boiling point, helps to avoid this issue [40]. Additionally, its surface free energy, which is close to graphene and substrate (47.7 mJ∙m^−2^), does not introduce any additional drawbacks. Moreover, EtOH and EG affect the rheology of inks by altering the molecular structure of carboxymethyl cellulose (CMC) in water. To demonstrate this, the rheological behavior of aqueous graphene inks was examined.

**Figure 4:**
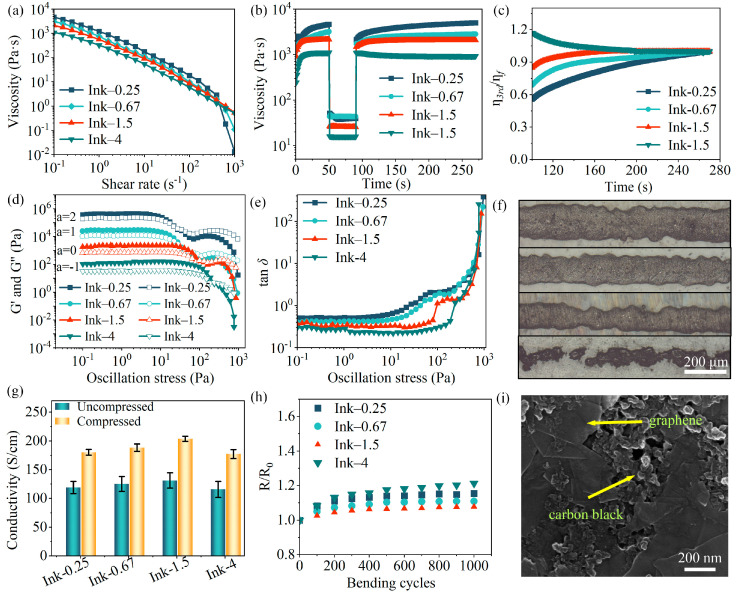
(**a**) Viscosity of ink as a function of shear rate; (**b**) thixotropy of ink; (**c**) percentage recovery of ink viscosity; (**d**) variation of storage modulus (G′), and loss modulus (G″) with shear stress, where the solid and hollow symbols stand for G′ and G″, respectively; (**e**) loss coefficient tan δ as a function of shear stress; (**f**) optical microscope images of thin lines of ink printed on PET substrate by passing through the opening of a 100 μm optical microscope images of fine lines printed by ink through screen openings on PET substrates, from top to bottom corresponding to Ink–0.25, Ink–0.67, Ink–1.5, and Ink–4, respectively; (**g**) electrical conductivity of the printed patterns before and after hot pressing; (**h**) change in the relative electrical resistance of the printed patterns during 1000 repetitive bending cycles at a bending angle of 120°; (**i**) top-view SEM image of the fine line printed with Ink–1.5.

As shown in Figure 4a, all inks exhibit high static viscosity and shear-thinning behavior. The viscosity of these inks decreases to ~10 Pa∙s at a shear rate of 100 s^−1^, allowing them to pass through the mesh under moderate conditions, which is extremely suitable for screen printing [29,41,42]. The change in static viscosity of the ink in relation to EtOH concentration is influenced by two contradictory factors: a decrease in viscosity due to the improved dispersion of graphene, as evidenced by the decreasing value of R_a_ with increasing EtOH content (Table S1), and an increase in viscosity caused by CMC. It is evident that the viscosity of inks ultimately depends on the latter factor (Figure S2a). This result also demonstrates that EtOH and EG have different mechanisms for increasing viscosity, primarily due to the disparity in the number of hydroxyl groups (–OH). EtOH competes with CMC for water interactions, and the highly favorable hydration of ethanol in the original aqueous phase displaces water from the vicinity of CMC molecules. Consequently, the average quantity of water molecules around the CMC molecules decreases, facilitating inter- and intra-chain bonding and resulting in the aggregation of CMC molecular chains [43]. Moreover, the relatively large number of hydroxyl groups in EG forms stronger hydrogen bonds with CMC molecules, thus minimizing its impact on the dispersion of CMC molecules and instead forming a dense network structure [44,45]. The observed approximate viscosities of all inks at high shear rates (>100 s^−1^) indicate that both EtOH and EG induce physical cross-linking of CMC molecular chains, which then resume their original elongated shape when subjected to higher shear stresses.

As shown in Figure 4b, the thixotropy of inks was evaluated by a three-interval thixotropy test (3ITT), which is divided into three intervals, i.e., static, high shear, and regeneration. The purpose is to document the deformation and recovery of the internal structure of inks that occurs during the printing process [11]. Figure S3 illustrates a typical screen printing process [46,47]. Initially, the ink is applied to the meshes of the stencil. Next, the squeegee moves across the stencil, pushing the ink through the meshes. Simultaneously, the substrate contacts the stencil to receive the ink. Finally, once the squeegee lifts upon completion of the printing process, the screen separates from the substrate, allowing for the recovery of any remaining ink on the substrate. The recovery index of the ink structure is typically defined as the ratio between the viscosity of the ink at the start of the regeneration interval and the viscosity of the static interval, which has a significant impact on the leveling and printing resolution of inks [48]. However, the recovery index of Ink–4 becomes impractical due to the spillover in the high-shear interval, resulting in a difference in ink quality between the regeneration and static intervals. Therefore, the viscosity recovery percentage (*η*_rec_), which divides the viscosity at any moment during the regeneration interval (*η*_3rd_) by the final viscosity (*η_f_*), was used to compare the recovery rates of different inks. A higher *η*_rec_ at a given moment indicates a more rapid recovery of ink viscosity. As shown in Figure 4c, *η*_rec_ demonstrates an upward trend with the rise in EG concentration, suggesting that the responsive damage inflicted upon the CMC network structure by high shear can be repaired more rapidly, which is primarily attributed to the contribution of hydrogen bonding between EG and CMC molecular chains. For example, the viscosity of Ink–1.5 returns to 90% within 10 s, allowing for high-resolution pattern printing. The *η*_rec_ of Ink–4, however, attains its peak at the onset of the regeneration interval and subsequently diminishes over time, probably due to the reduced dispersion of graphene, which has a significantly larger Ra than the other inks.

Figure 4d demonstrates the viscoelastic effect of inks during the stress sweep step by performing oscillatory rheology measurements. The data points in the graph were vertically shifted by multiples of 10^a^ to prevent overlap of the plotted elements. As a viscoelastic material, the ink exhibits strain in response to shear stress. Within the linear viscoelastic region (LVE), the ink structure undergoes reversible changes irrespective of the shear stress. However, once the strain surpasses the LVE, the deformation of the ink structure becomes irreversible. The storage modulus (G′) characterizes the elasticity of the ink, while the yield stress (τ*_y_*) represents the structural strength of the ink and signifies the maximum stress that can be exerted without causing damage to its structure. Furthermore, the flow stress (τ*_f_*) denotes the minimum stress required for the ink to flow, indicating the difficulty of passing the ink through the screen [48]. Taking ink–1.5 as an example, in the LVE, where the shear stress is below 9.8 Pa, the ink can withstand external stress without damage to its structure and can also recover elastically to its initial state under any stress. As the shear stress increases, both G′ and G″ decrease gradually when the shear stress exceeds 9.8 Pa. However, G′ remains higher than the value of G″, indicating a weakening and gradual rupture of the ink structure. When the shear stress exceeds 99.8 Pa (G″ = G′), the ink transitions from a solid-like behavior to a liquid-like behavior and starts to flow. The viscoelastic parameters of inks are shown in Table 1. G′, τ*_y_*, and τ*_f_* exhibit a significant correlation with the ratio of EG to EtOH (from 0.25 to 4). Specifically, G′ decreases from 4208 Pa to 1109 Pa, τ*_y_* increases from 3.3 Pa to 11.2 Pa, and τ*_f_* increases from 27.4 Pa to 240.3 Pa. Regarding this, the aggregation of CMC molecules induced by the strong hydration of EtOH forms a secondary molecular structure with high stiffness and energy storage modulus (G′) in the small strain range (Figure S2d). Nevertheless, the low concentration of CMC employed only as an additive implies a low cross-linking density, and the negative charge of the carboxyl ions (–COO^−^) on the CMC molecule repels the secondary molecular structure. Consequently, CMC molecules exhibit an aggregated structure at small scale but a loosely connected structure at large scale, in which the molecular chains are susceptible to slipping and reorganizing, making them vulnerable to irreversible deformation and flow at low τ*_y_* and τ*_f_* (Figure S2d). Furthermore, the recovery rate of structure is slower (Figure S2b,c). In contrast, a higher concentration of EG implies a stronger hydrogen bonding interaction with the carboxyl (–COOH) and hydroxyl (–OH) groups on the CMC molecule, which induces the CMC molecule to form a denser and more stable network structure. In this way, the structural strength of the ink is enhanced, although the difficulty of printing increases due to an increased τ*_f_* [42]. These findings are further supported by the alteration in the loss coefficient tan*δ* (decreasing from 0.504 to 0.298), an indicator of the crosslink density and the strength of interactions within inks (Figure 4e). 

The effect of ink rheology on printing resolution was studied by printing lines of various widths (Figure 3g, Figures S4 and S5). The printing resolution is determined by the minimum width of a complete short line that can be obtained by screen printing. As shown in Figure 3g, although all inks can pass through a stencil opening with a width of 100 μm, only Ink–0.25, Ink–0.67, and Ink–1.5 can create continuous and thin lines with actual widths of 151, 137, and 109 μm. This is consistent with the rheological results of the inks, where the short viscosity recovery time of inks with low thixotropy (Ink–1.5) avoids short-circuiting caused by wide spreading of the ink on the substrate, which is favorable for scenarios requiring precision printing. However, excessive elasticity affects the leveling of inks, which disrupts the uniformity and even the continuity of the lines, resulting in the interruption of the lines printed by Ink–4 (Figure 3g). Therefore, considering the preceding analysis, we believe that Ink–1.5 represents an aqueous graphene conductive ink with excellent printability. In addition, the clarity and integrity of the patterns printed by Ink–1.5 on the substrates, such as PET, PI, and paper, demonstrate the outstanding adaptability of Ink–1.5 to a wide range of substrates (Figure S4).

Next, the influence of ink properties on the electrical characteristics of printed patterns was investigated. The electrical conductivity of printed patterns increases with the concentration of EG and peaks at 1.31 × 10^4^ S∙m^−1^ when the ratio of EG to EtOH reaches 1.5 (Figure 4g). The aggregation of CMC molecules induced by EtOH may increase the distance among conductive fillers, thus leading to a reduction in the electrical conductivity of Ink–0.25 and Ink–0.67. The low electrical conductivity of Ink–4 may be attributed to a recovery time that is insufficient to adequately compensate for printing defects. According to previous reports, the conductivity of printed patterns can be enhanced through post-processing, so dried patterns were passed through a roller press at a temperature of 80 °C. Interestingly, both the thickness and sheet resistance (R_S_) of printed patterns decrease. Furthermore, it can be inferred that the flatness of printed patterns increases based on the shortened error bars (Figure 4g) [29]. The conductivity of Ink–1.5 increases to 2.04 × 10^4^ S∙m^−1^ after hot pressing, at which time the thickness and R_S_ of the pattern were 5 μm and 9.8 Ω∙sq^−1^, respectively. Additionally, the R_S_ of printed patterns can be further reduced by increasing the passes of printing, while the electrical conductivity may decrease due to defects resulting from an increase in the thickness. Nevertheless, all printed patterns, independent of thickness, reach the 5B class of adhesion strength, except for those of 30-μm thickness on the PI foil (Figure S5). Notably, the change in normalized resistance of the pattern printed by Ink–1.5 is less than 1.08% after 1000 bending cycles at an angle of 120°, indicating its excellent flexibility (Figure 4h), which is attributed to the dense conductive network formed by graphene and carbon black (Figure 4i and Figure S6). Therefore, it is believed that the aqueous graphene ink developed here can produce flexible devices with excellent mechanical characteristics. A comparison of our work with other graphene-based conductive inks is presented in Table S2. It can be concluded that aqueous graphene ink (Ink–1.5) developed here with green and remarkable conductive properties, benefiting from its high loading of graphene and short-time post-processing with low temperature, can be integrated with the roll-to-roll (R2R) process for the fabrication of flexible electronic devices with excellent mechanical characteristics on a variety of substrates.

### 3.3. Electrochemical Performance of Micro-Supercapacitors

**Figure 5:**
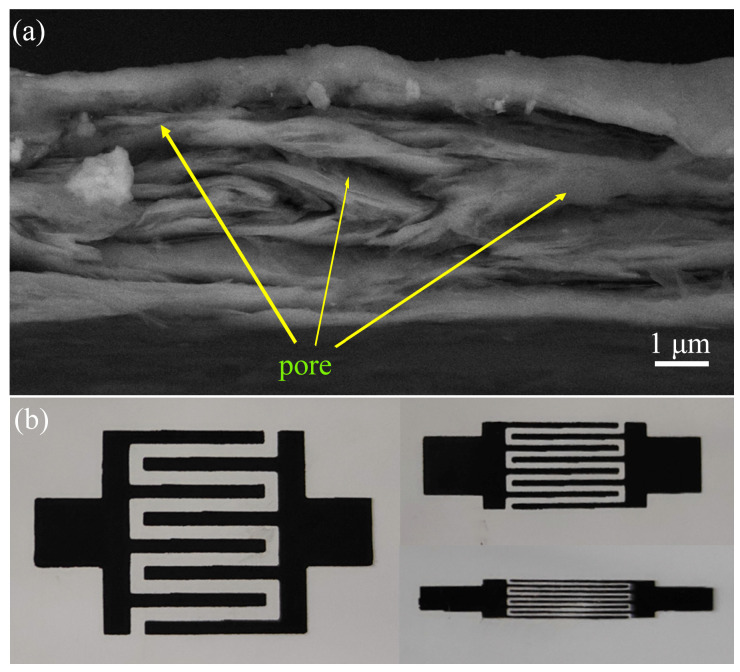
(**a**) A representative cross-sectional SEM image of MSC. (**b**) A digital photograph of MSC_1000_, MSC_500_, and MSC_200_.

Due to the excellent charge storage capability of graphene and the high conductivity of the printed pattern, Ink–1.5 with high printing resolution has been utilized to print planar micro-supercapacitors (MSCs) without metal collectors. The representative cross-sectional SEM image of the MSC_200_ microelectrode depicts the thickness of the laminar structure, composed of graphene/carbon black on PET substrate, to be approximately 5 μm. (Figure 5a). Figure S7 shows that the electrode contains mesoporous material with a diameter of 2–50 nm. Importantly, the porous morphology of the electrode increases the accessible surface area of the electrolyte and facilitates the diffusion of the electrolyte within the electrode, which is crucial in enhancing the electrochemical performance of MSCs [49,50]. Recent studies have shown that the power capacity of MSCs can be significantly improved by reducing electrode width and separation [16,17,25]. Therefore, to examine the influence of geometrical configuration on the electrochemical properties of MSCs, three types of MSCs were fabricated with varying finger width and interspace dimensions (1000, 500, and 200 μm), and the corresponding microdevices were denoted as MSC_1000_, MSC_500_, and MSC_200_, as shown in Figure 5b. The specific size parameters of the MSC electrodes are shown in Figure S8. The screen-printed MSC configuration comprises eight fingers that form an interdigitated structure. The effective areas of MSC_1000_, MSC_500_, and MSC_200_ are 1.35, 0.675, and 0.27 cm^2^, respectively.

**Figure 6:**
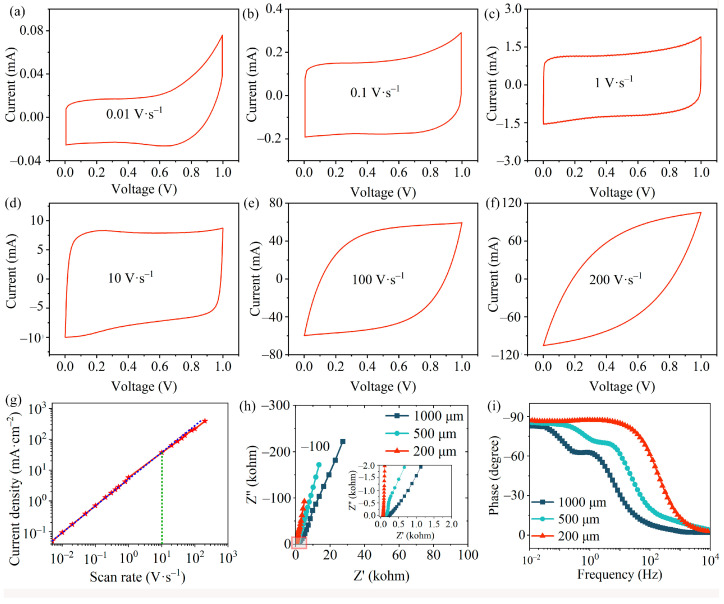
(**a**–**f**) CV curves of MSC_200_ at different scan rates of 0.01, 0.1, 1, 10, 100, and 200 V∙s^−1^. (**g**) The relationship of discharge current density varies with the scan rate for MSC_200_. (**h**) Nyquist plot of MSC_200_, MSC_500_, and MSC_1000_. (**i**) The relationship of impedance phase angle varies with frequency for the three MSCs.

Figure 6a–d and Figures S9–S10 illustrate the CV tests conducted to investigate the power capability of MSCs at scan rates of 0.005–200 V∙s^−1^. The three MSCs exhibit a nearly rectangular CV shape at lower scan rates, aligning with expectations for double-electric layer materials. This suggests that screen-printed graphene-based interdigitated microelectrode structures can provide efficient and reversible electrochemical properties. Remarkably, double-electric layer capacitance (EDLC) behavior can be maintained at an impressive scan rate of 100 V∙s^−1^ for MSC_200_ (Figure 6e). A linear relationship between discharge current and scan rate persists up to at least 10 V∙s^−1^ (Figure 6g). MSC_200_ allows rapid charging and discharging at an extremely high scan rate of up to 200 V∙s^−1^ while maintaining outstanding capacitance, showcasing high transient power characteristics (Figure 6f) [51]. This is much higher than the maximum scan rates of the MSC_500_ (10 V∙s^−1^) and MSC_1000_ (1 V∙s^−1^) (Figures S9 and S10). Such enhanced rate performance exceeds that of traditional supercapacitors by at least two orders of magnitude and outperforms any previously reported values for fully printed graphene-based micro-supercapacitors [12,16,51,52].

To better understand the mechanism by which the geometric configuration of electrodes impacts the electrochemical performance of MSCs, the EIS of graphene-based MSC_200_, MSC_500_, and MSC_1000_ was measured. As shown in Figure 6h, smaller finger widths and spacing significantly reduce the equivalent series resistance (ESR) of MSCs, which is acquired from the intercept of EIS with the real axis at high frequencies. ESR decreases from 196.6 Ω to 63.4 Ω with the reduction in finger width and spacing from 1000 µm to 200 µm. Moreover, the Nyquist plot of MSC_200_ exhibits a steeper slope at low frequencies, approaching the Y-axis more closely than those of MSC_500_ and MSC_1000_, suggesting a better EDLC performance for interdigitated structures with smaller finger widths and spacing. The slope of the line at low frequencies can be interpreted as the inverse of the Warburg impedance, signifying the constraint on ion diffusion between the electrolyte and electrodes [53]. Therefore, the reduction in both ESR and ion transport limitations leads to the high power capability of the MSC_200_. To conduct a comprehensive analysis of the EIS measurements, the characteristic frequency (*f*_0_) and their corresponding time constants (*τ*_0_ = 1/*f*_0_) of the three MSCs at a phase angle of –45° were examined. The phase angle, depicted as a frequency-dependent function, is shown in Figure 6i. *τ*_0_ represents the minimum time for MSCs to release all their energy with an efficiency greater than 50% [54]. The characteristic frequency of MSC_200_ is 210 Hz, which is much higher than that of MSC_500_ (21.5 Hz) and MSC_1000_ (5.6 Hz). The corresponding *τ*_0_ for MSC_200_ is 4.8 ms. In contrast, the values of *τ*_0_ for MSC_500_ and MSC_1000_ are much larger, 46.5 and 179 ms, respectively. The low *τ*_0_ values again reveal the fast ion diffusion behavior and superior power responsiveness of MSC_200_.

**Figure 7:**
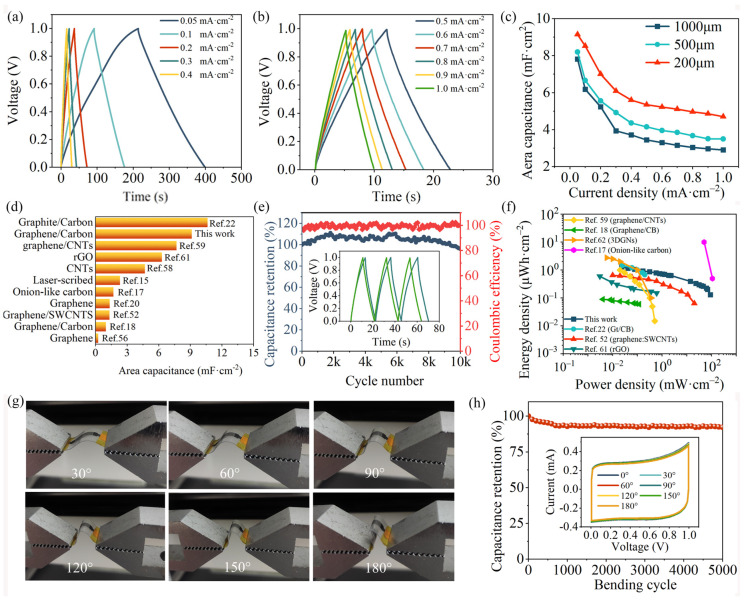
GCD curves at varying current densities of (**a**) 0.05–0.4 mA∙cm^−2^ and (**b**) 0.5–1.0 mA∙cm^−2^. (**c**) Area capacitance of the three MSCs varying current density of 0.05–1.0 mA∙cm^−2^. (**d**) Comparison of area-specific capacitance of this study and other reported printed MSCs. (**e**) Cycling performance of MSC_200_ at the current density of 0.5 mA∙cm^−2^. (**f**) Ragone plot on energy density and power density. (**g**) Photographs of MSC_200_ at various bending angles. (**h**) Capacitance retention of MSC_200_ during 5000 bending cycles. Inset: CV curves obtained at various bending angles using a scan rate of 200 mV∙s^−1^ [15,17,18,20,22,52,56,58,59,61,62].

The GCD results of MSCs at the current density ranging from 0.05 to 1 mA∙cm^−2^ are shown in Figure 7a–b and Figure S11. Regardless of the width and gap distance of the fingers, the approximate symmetric curves indicate the excellent capacitive behavior of graphene-based MSCs prepared by screen printing, in accordance with the CV results. An increase in current density and scan rate results in a restricted diffusion effect, which prevents charge storage in some areas of the active surface, so the area- and volume-specific capacitance of the MSCs gradually decreases (Figure 7c, Figures S12 and S13) [54]. Importantly, specific capacitance, which increases with narrower finger width and spacing, exhibits a more slowly decreasing trend with higher current density and scan rate. For instance, the area- and volumetric-specific capacitances of MSC_200_ are 9.15 mF∙cm^−2^ (Figure 7c) and 13.07 F∙cm^−3^ (Figure S13a) at a current density of 0.05 mA∙cm^−2^, respectively. Even when subjected to a high current density of 1.0 mA∙cm^−2^, MSC_200_ can still exhibit impressive area- and volumetric-specific capacitances of 4.7 mF∙cm^−2^ and 6.71 F∙cm^−3^, respectively, highlighting its outstanding rate capability. In contrast, the specific capacitance of MSC_500_ and MSC_1000_ can only be retained by 43% and 37% when the current density increases from 0.05 mA∙cm^−2^ to 1.0 mA∙cm^−2^. The exceptional electrochemical performance of MSC_200_ is mainly attributed to narrower finger width and distance that effectively reduce the ion diffusion path in the inner region of interdigitated electrodes and maximize the available electrochemical surface area [55,56]. The area-specific capacitance of fully printed MSC_200_ outperforms that of most other reported carbon-based MSCs (Figure 7d), which are prepared by printing [18,20,22,52,57,58,59,60] mask spraying [61], electrophoretic deposition [17], and laser-induced or cutting techniques [15,62,63], especially considering the absence of metal collectors in the MSC_200_. Furthermore, such fully printed MSC_200_ demonstrates remarkable electrochemical stability with a high capacitance retention of 91.6% and a coulombic efficiency (CE) close to 100% during 10,000 cycles at a current density of 0.5 mA∙cm^−2^ (Figure 7e). The durability and reliability of MSC_200_ were further verified by performing 30,000 cycles at a high scan rate of 10 V·s^−1^. As illustrated in Figure S18, MSC_200_ retained 84.6% of its initial capacitance after 30,000 cycles, demonstrating excellent cycling stability during rapid charge and discharge (Figure S14). The area energy and power density of MSC_200_, based on different scan rates, along with the results of other carbon-based MSCs, are shown in the Ragone plot (Figure 7f). MSC_200_ delivered an area energy and power density of 1.30 µWh∙cm^−2^ and 0.023 mW∙cm^−2^ at a scan rate of 5 mV∙s^−1^. Remarkably, MSC200 exhibits an exceptionally high power density of 89.9 mW∙cm^−2^, surpassing the majority of carbon-based MSCs. Although MSCs based on onion-like carbon nanomaterials have demonstrated record-high area performance, onion-like carbon nanomaterials stem from nanodiamond powders thermally annealed at temperatures up to 1700 °C [17]. 

The effect of electrode thickness on the electrochemical properties of MSC_200_ was also investigated. The results are presented in Figures S15–S17. As depicted in Figures S15–S16, as the electrode thickness increases, the near-rectangular shape of the CV curves can only be preserved at lower scan rates, such as 10 V·s^−1^ for a thickness of 7 μm and 5 V·s^−1^ for 10 μm. The areal capacitance reaches its peak at a thickness of 7 μm, while the volumetric capacitance, power density, and energy density decrease with further increases in thickness. This is because greater thickness results in more active material per unit area; however, it also lengthens the ion diffusion path, thereby reducing the effective surface area of the electrodes (Figure S17).

To further evaluate its potential application in wearable/portable devices, the electrochemical performance of MSC200 was investigated at various bending angles from 0° to 180° (Figure 7g). As shown in Figure 7h, it is worthy noted that all the CV curves remain almost identical and 92.2% of the initial capacitance can be maintained after 5000 bending cycles with a bending angle of 120°, emphasizing outstanding mechanical flexibility and durable stability to store charge of the fully printed MSCs, which is credited to the synergistic effects of aqueous conductive ink and hydrogel-polymer electrolyte, including: (1) excellent adhesion of aqueous polyurethane to the PET substrate, (2) dense conductive network consisting of a mesoporous structure formed by graphene with dispersed carbon black acting as linkers, (3) outstanding flexibility of the solid electrolyte, and (4) superior wettability of hydrophilic components in the interdigitated electrodes with hydrogel-polymer electrolytes. Impressively, modular MSCs (5S × 5P) can be used as the power source to drive a wearable Joule heater, allowing it to produce uniform heating (Figure S18). Given the frequent exposure to moisture and salt, the waterproofing and weather resistance of wearable supercapacitors are crucial. Effective encapsulation is a key measure to protect MSCs from environmental influences. The MSCs were encapsulated by pouring polyurethane (PU) over them and curing at 80 °C. Subsequently, they were sewn into a garment to simulate a real washing process. After drying, the CV curves of the MSCs were measured. The similarity of the CV curves before and after washing demonstrates the effectiveness of the encapsulation (Figure S19).

## 4. Conclusions

In summary, we have developed the scalable production of aqueous graphene conductive inks with environmentally friendly and excellent printability by a sand-mill method for high-resolution screen printing of solid-state flexible micro-supercapacitors. In particular, such a method of preparing high concentration inks without expensive equipment and harsh post-treatments is representative of simplicity, efficiency, and cost-effectiveness, which makes the inks suitable for the roll-to-roll (R2R) process for one-step printing highly conductive patterns on a variety of substrates. The tuned rheology of graphene inks achieved by varying the ratio of ethylene glycol to ethanol in the solvent results in a uniform pattern with a resolution of ~100 μm and superior electrical conductivity of 2.04 × 10^4^ S∙m^−1^, as well as extraordinary mechanical properties. The screen-printed flexible MSCs demonstrate a high area-specific capacitance of 9.15 mF∙cm^−2^, an area energy density of 1.30 µWh∙cm^−2^, and an ultra-high area power density of 89.9 mW∙cm^−2^. Furthermore, the fully printed MSCs exhibit durable performance with 91.6% of the initial capacitance and a coulombic efficiency of approximately 100% after 10,000 cycles, as well as an outstanding mechanical characteristic that maintains 92.2% of the initial capacitance over 5000 bending cycles. These results indicate that the aqueous graphene conductive inks reported here hold great promise for miniaturized, high-performance, wearable/portable printed electronics.

## Figures and Tables

**Table 1:** Viscoelastic parameters of inks obtained by the stress sweep test.

	Ink–0.25	Ink–0.67	Ink–1.5	Ink–4
G′ (Pa)	4208	2941	2048	1109
G″ (Pa)	2118	1246	715	330
Tan*δ* (G″/G′)	0.504	0.424	0.349	0.298
τ*_y_* (Pa)	3.3	6.7	9.8	11.2
τ*_f_* (Pa)	27.4	43.2	99.8	240.3

## Data Availability

The original contributions presented in the study are included in the article/supplementary material, further inquiries can be directed to the corresponding author.

## Supplementary Materials

The following supporting information can be downloaded at: https://www.mdpi.com/article/10.3390/ma17164021/s1, Figure S1: Survey XPS of pristine graphite and graphene; Table S1: The physical and chemical parameters of different materials; Figure S2: (a) Viscosity of CMC in water–alcohol solvents as a function of shear rate. (b) Viscosity of CMC in water–alcohol solvents as a function of time in a 3ITT test. (c) Viscosity recovery of CMC in water–alcohol solvents during the third stage of the 3ITT test. (d) Stress oscillation sweep results of CMC in water–alcohol solvents. Solid and hollow symbols represent the storage modulus (G′) and loss modulus (G″), respectively; Figure S3: Schematic diagram of a typical screen printing process. (a) The ink is first spread over the mesh openings of the screen. (b) The squeegee then moves across the screen, forcing the ink through the openings, while the substrate contacts the screen to receive the ink. (c) Finally, when the squeegee lifts, the screen separates from the substrate, and the ink left on the substrate recovers; Figure S4: Optical image of (a) a 325-mesh screen printing stencil. (b–d) Optical images of printed lines with Ink–1.5 on PET foil, PI foil, and paper, respectively; Figure S5: (a) Sheet resistance and (b) conductivity of printed patterns on PET as a function of thickness, respectively. Adhesion strength of printed patterns on PET substrate with thicknesses of (a) 10 μm, (b) 20 μm, and (c) 30 μm. Adhesion strength of printed patterns on the PI substrate with thicknesses of (d) 10 μm, (e) 20 μm, and (f) 30 μm; Table S2: Comparison of our work with other graphene-based conductive inks; Figure S6: Top-view SEM image of the fine line printed with Ink–1.5; Figure S7: (a) Isothermal adsorption curve and (b) pore size distribution of the electrode; Figure S8: Electrode geometries of three graphene-based MSCs, namely MSC_1000_, MSC_500_, and MSC_200_. The table presents their specific parameters; Figure S9: CV curves of MSC_500_ at different scan rates of 0.01, 0.1, 1, and 10 V·s^−1^; Figure S10: CV curves of MSC_1000_ at different scan rates of 0.01, 0.1, and 1 V·s^−1^; Figure S11: GCD curves of MSC_500_ at different current densities of (a) 0.05–0.4 mA·cm^−2^ and (b) 0.5–1.0 mA·cm^−2^. GCD curves of MSC_1000_ at different current densities of (c) 0.05–0.4 mA·cm^−2^ and (d) 0.5–1.0 mA·cm^−2^; Figure S12: (a) Areal capacitance and (b) volumetric capacitance of MSCs based on CV curves; Figure S13: (a) Volumetric capacitance of MSC_200_ based on GCD curves. (b) Capacitance retention of MSC_200_ at different current densities relative to 0.05 mA·cm^−2^; Figure S14: Cycling performance of MSC_200_ at the scan rate of 10 V∙s^−1^. Figure S15: (a–d) CV curves of MSC_200_ with a thickness of 7 μm at different scan rates of 0.1, 1, 10, and 50 V·s^−1^. Figure S16: (a–d) CV curves of MSC_200_ with a thickness of 10 μm at different scan rates of 0.1, 1, 5, and 10 V·s^−1^; Figure S17: (a) Areal capacitance, (b) volumetric capacitance, and (c) Ragone plot on energy density and power density of MSC_200_ with thicknesses of 5, 7, and 10 μm as a function of scan rate. Table S3: Comparison of our work with the state-of-the-art MSCs based on carbon materials. Figure S18: The modular MSCs power a wearable heater for uniform heating. Figure S19: CV curves of PU-encapsulated MSC_200_ at 100V·s^−1^ scan rate before and after washing.
