# Supplementary material for: Planar Micro-Supercapacitors with High Power Density Screen-Printed by Aqueous Graphene Conductive Ink
Source: Materials (Basel). 2024 Aug 13;17(16):4021. doi: 10.3390/ma17164021 (PMC11356036; doi:10.3390/ma17164021)
Supplement: Supplementary file 1 [file materials-17-04021-s001.zip › Supplementary Materials.pdf]

Electronic Supplementary Information (ESI)

Planar Micro-Supercapacitors with High Power Density Screen-Printed by Aqueous Graphene Conductive Ink

Youchang Wang [1,2], Xiaojing Zhang [1,2,], Yuwei Zhu [1,2], Xiaolu Li [1,2] and Zhigang Shen [1,2]*

[1] Beijing Key Laboratory for Powder Technology Research and Development, Beihang University, Beijing 100191, China

[2] School of Aeronautic Science and Engineering, Beihang University, Beijing 100191, China

E-mail: zhangxiaojing@buaa.edu.cn

Calculations: The capacitance were calculated from the CV curves by integrating the discharge portion using the following equation:

$$C = \frac{1}{\nu(V_f - V_i)} \int_{V_i}^{V_f} I(V)dV$$

where V_f and V_i are the integration voltage limits, ν is the scan rate (V·s $^{-1}$), and $I(V)$ is the discharge current (A). The capacitance were also calculated from the GCD curves using the following equation:

$$C = \frac{I \times \Delta t}{\Delta V}$$

where I is the discharge current (A), Δt is the discharge time (s), and ΔV is the discharge potential interval (V). Based on the area and volume of MSCs, the area and volumetric specific capacitance of MSCs were calculated using the following equations:

$$C_A = C/A$$

$$C_V = C/V$$

where C_A (mF·cm^{-2}) and C_V (F·cm^{-3}) are the area and volumetric specific capacitance of MSCs, respectively. A and V are the total area and volume of MSCs (including the interdigitated electrodes and the gaps between them), respectively. Additionally, the volumetric energy density (E_A) and power density (P_A) of MSCs were calculated using the following equations:

$$E_A = \frac{1}{2} \times C_A \times \frac{(\Delta V)^2}{3.6}$$

$$P_A = \frac{E}{\Delta t} \times 3600$$

where E_A is the area energy density (μWh·cm^{-2}), P_A is the area power density (mW·cm^{-2}), C_A is the area specific capacitance, ΔV is the discharge potential range (V), and $\Delta t$is the discharge time (s).

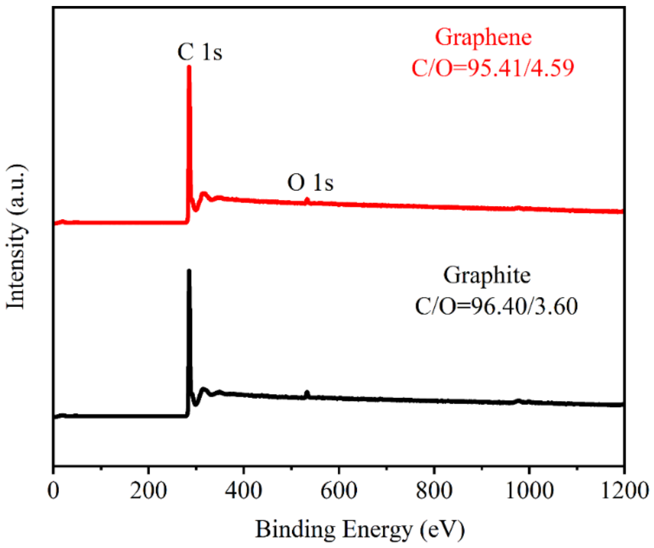

Figure S1. Survey XPS of pristine graphite and graphene.

Table S1. The physical and chemical parameters of different materials.

Material	Surface free energy (mJ·m^{-2})	Boiling point (°C)	Viscosity (mPa·s)	$\delta_D{}^*$ (MPa$^{1/2}$)	$\delta_P{}^*$ (MPa$^{1/2}$)	$\delta_H{}^*$ (MPa$^{1/2}$)	$R_a{}^*$ (MPa$^{1/2}$)
H_2O	72.8	100.0	1.0	15.5	16	42.3	35.6
EtOH	22.1	78.4	1.2	15.8	8.8	19.4	12.5
EG	47.7	197.3	16.1	17	11	26	18.5
H_2O/EtOH	/	/	/	15.7	12.0	29.5	22.5
H_2O/EG	/	/	/	16.2	13.6	34.6	27.5
E-G-0.25*	/	/	/	15.8	12.3	30.4	23.3
E-G-0.67*	/	/	/	15.9	12.6	31.3	24.2
E-G-1.5*	/	/	/	16.0	12.9	32.3	25.2
E-G-4*	/	/	/	15.9	13.8	35.1	28.1
Graphene	46.7	/	/	18	9.3	7.7	/

δ_D, δ_P, δ_H: dpersive, polar, and hydrogen-bonding solubility parameters.

R_a: solubility radius.

E-G-x : mixture solvent with a ratio of EG to EtOH, where x is 4, 1.5, 0.67, and 0.25.

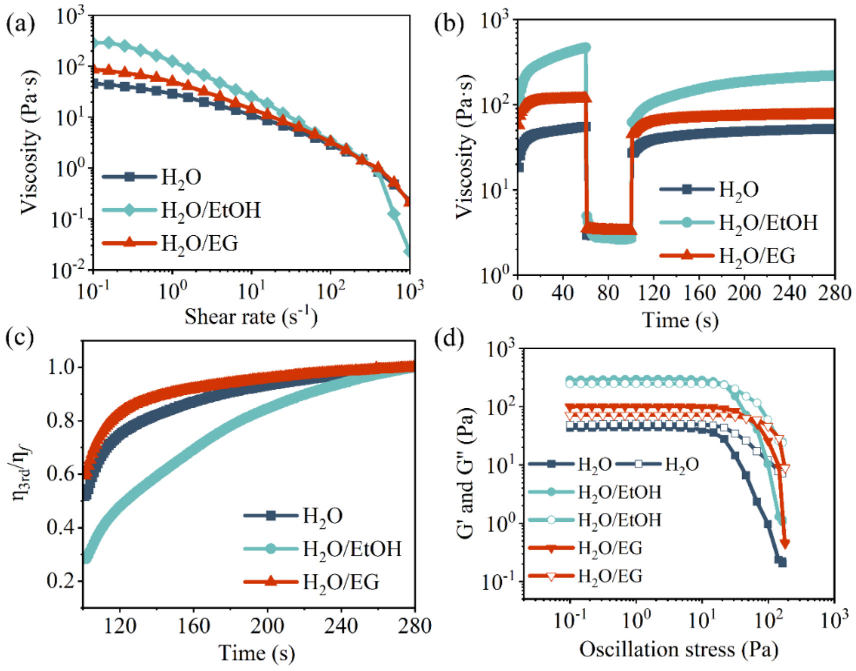

Figure S2. (a) Viscosity of CMC in water-alcohol solvents as a function of shear rate. (b) Viscosity of CMC in water-alcohol solvents as a function of time in a 3ITT test. (c) Viscosity recovery of CMC in water-alcohol solvents during the third stage of the 3ITT test. (d) Stress oscillation sweep results of CMC in water-alcohol solvents. Solid and hollow symbols represent the storage modulus (G′) and loss modulus (G″), respectively.

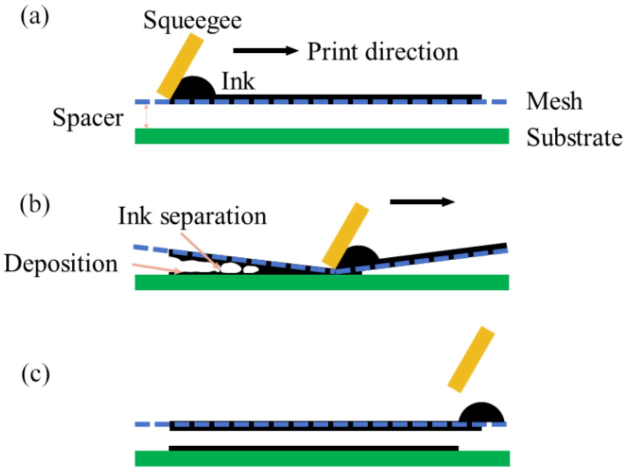

Figure S3. Schematic diagram of a typical screen printing process. (a) The ink is first spread over the mesh openings of the screen. (b) The squeegee then moves across the screen, forcing the ink through the openings, while the substrate contacts the screen to receive the ink. (c) Finally, when the squeegee lifts, the screen separates from the substrate, and the ink left on the substrate recovers.

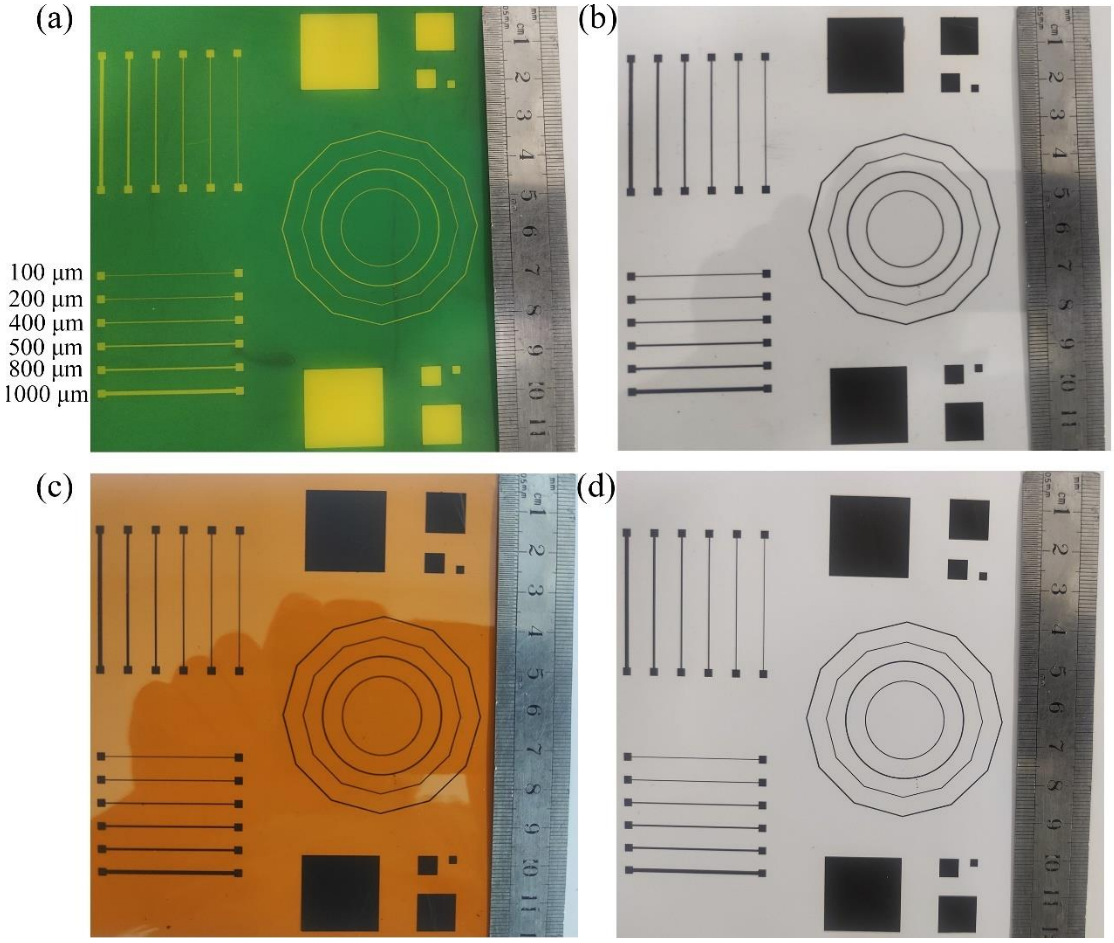

Figure S4. Optical image of (a) a 325-mesh screen printing stencil. (b~ d) Optical images of printed lines with Ink-1.5 on PET foil, PI foil and A4 paper, respectively.

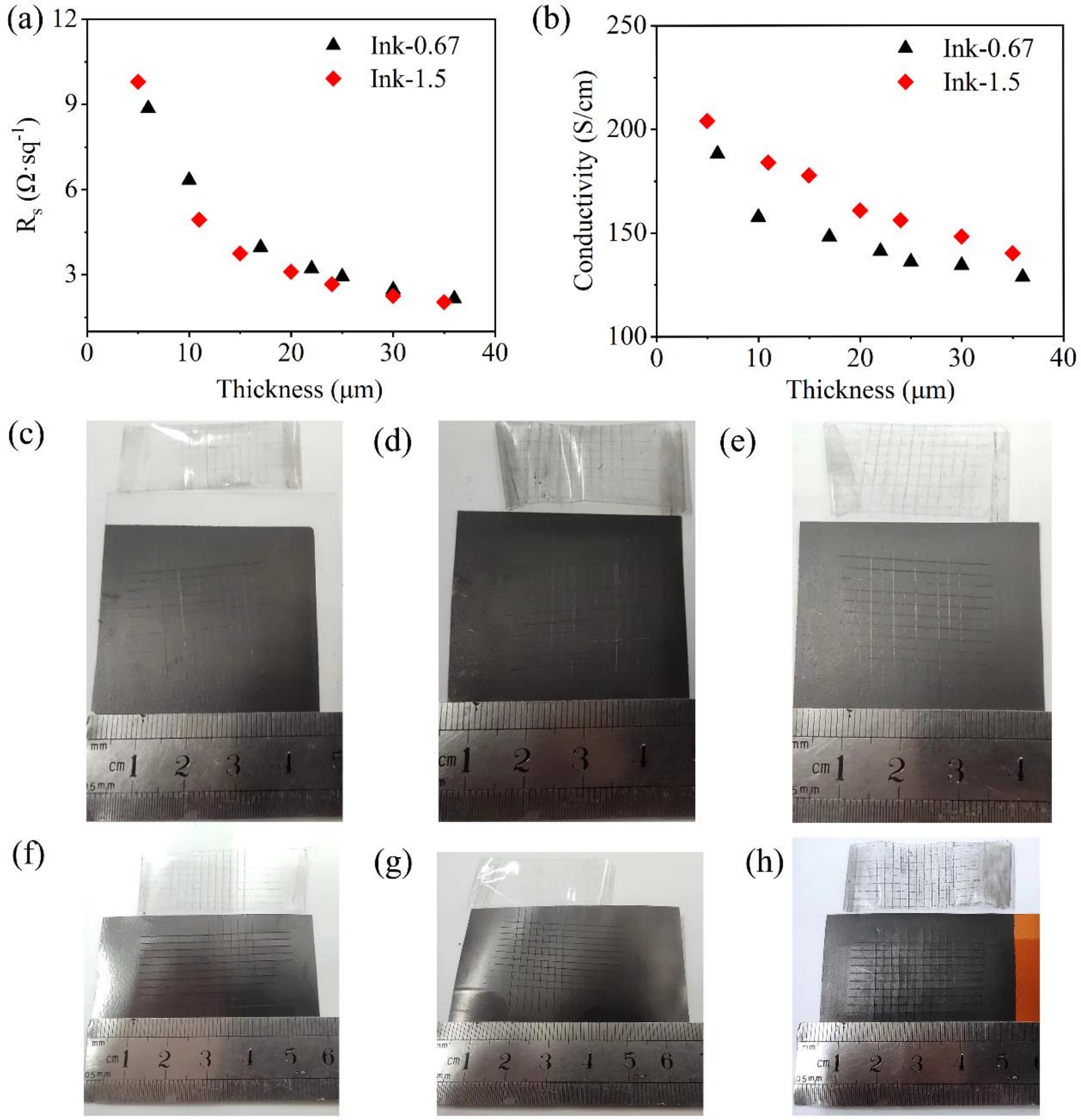

Figure S5. (a) Sheet resistance and (b) conductivity of printed patterns on PET as a function of thickness, respectively. Adhesion strength of printed patterns on PET substrate with thicknesses of (a) 10 μm, (b) 20 μm, (c) 30 μm. Adhesion strength of printed patterns on the PI substrate with thicknesses of (d) 10 μm, (e) 20 μm, (f) 30 μm.

Table S2. Comparison of our work with other graphene-based conductive inks

Concentration (mg/ml)	Solvent	Substrate	Annealing temperature (°C)	Annealing time (min)	Electrical conductivity (S/m)	Ref.
80	Terpineol	PI	300	30	~1.0×10^4	1
110-115	H_2O/BDO/IPA	PI	300	90	8.7×10^4	2
			50	30	9.41×10^3	
100	H_2O	Glass	300	40	2×10^4	3
70	Cyrene	Paper	100	300	3.7×10^4	4
52	DPM	PET	Photonic annealing	/	7.29×10^3	5
170	DBE	PET	150	16	2.15×10^4	6
47.5	Ethanol/terpineol	PET	225	120	1.49×10^4	7
200	Ethanol/terpineol	PI	300	30	1.86×10^4	8
130	H_2O/EtOH/EG	PET	80	15	2.04×10^4	This work

BDO: 2, 3-Butanediol, IPA: isopropanol, EtOH: ethanol, EG: ethylene glycol, DBE: Dibasic Esters, DPM: methyl ether of dipropylene glycol

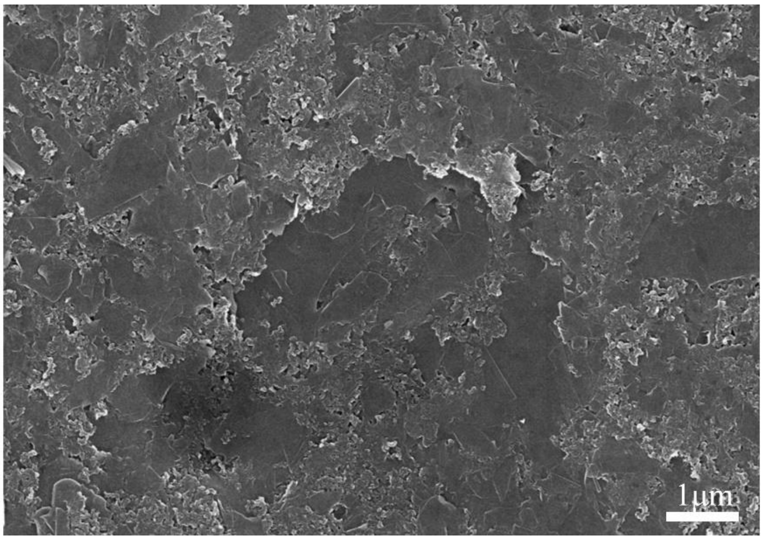

Figure S6. Top-view SEM image of the fine line printed with Ink-1.5.

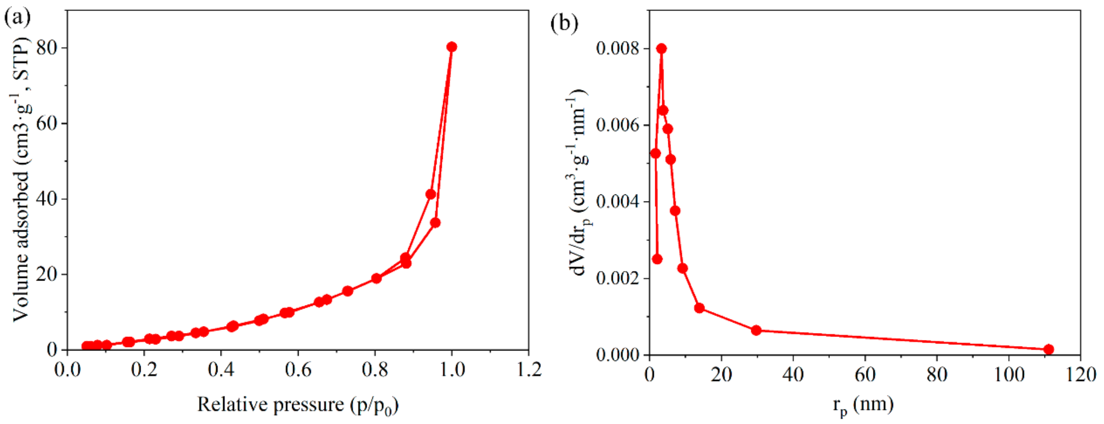

Figure S7. (a) Isothermal adsorption curve and (b) Pore size distribution of the electrode

MSC	MSC_{1000}	MSC_{500}	MSC_{200}
**Length, *l* (mm)**	9	9	9
**Width, *w* (μm)**	1000	500	200
**Interspace, *i* (μm)**	1000	500	200
**Area (cm^{-2})**	1.35	0.675	0.27

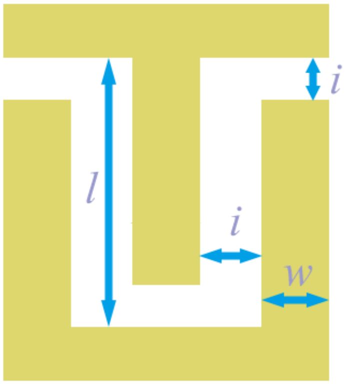

Figure S8. Electrode geometries of three graphene-based MSCs, namely MSC_{1000}, MSC_{500}, and MSC_{200}. The table presents their specific parameters.

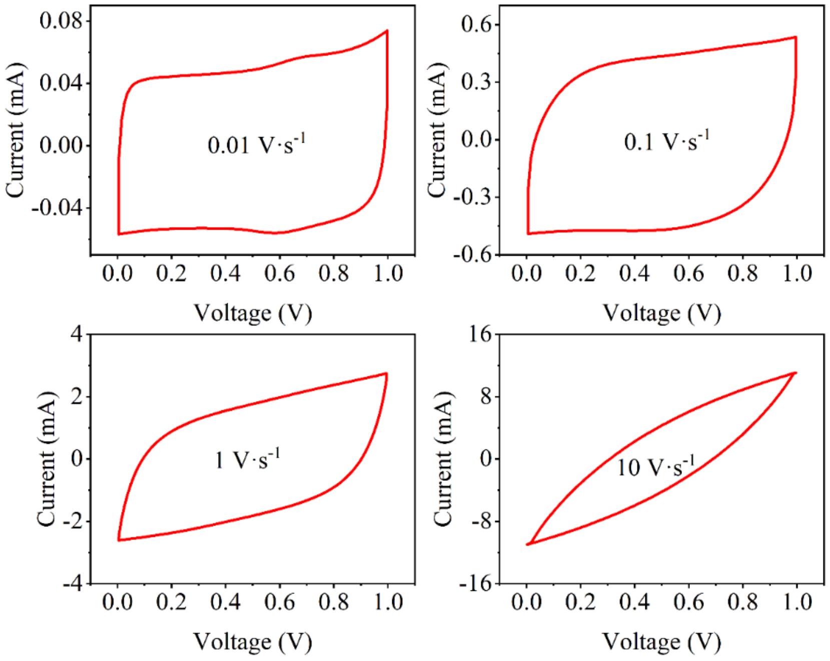

Figure S9. CV curves of MSC_{500} at different scan rates of 0.01, 0.1, 1, and 10 $V{\cdot}s^{-1}$.

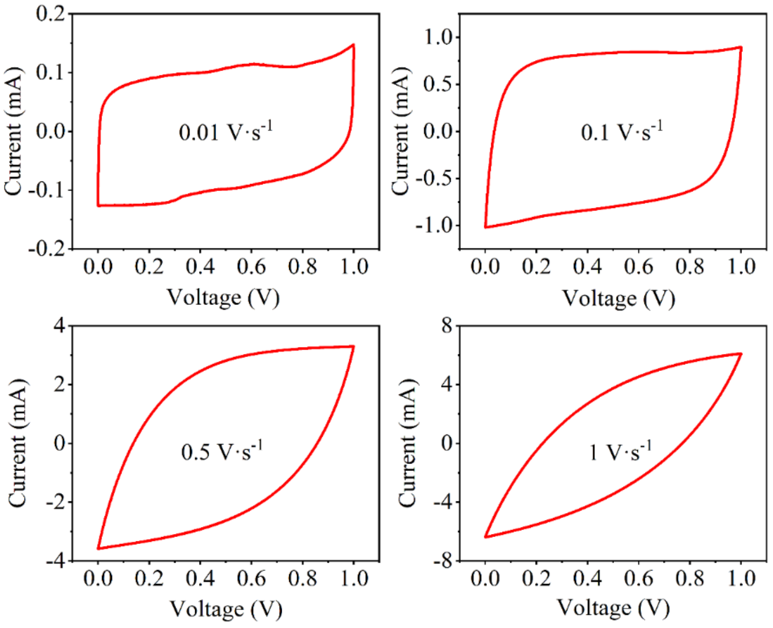

Figure S10. CV curves of MSC_{1000} at different scan rates of 0.01, 0.1, and 1 $V \cdot s^{-1}$.

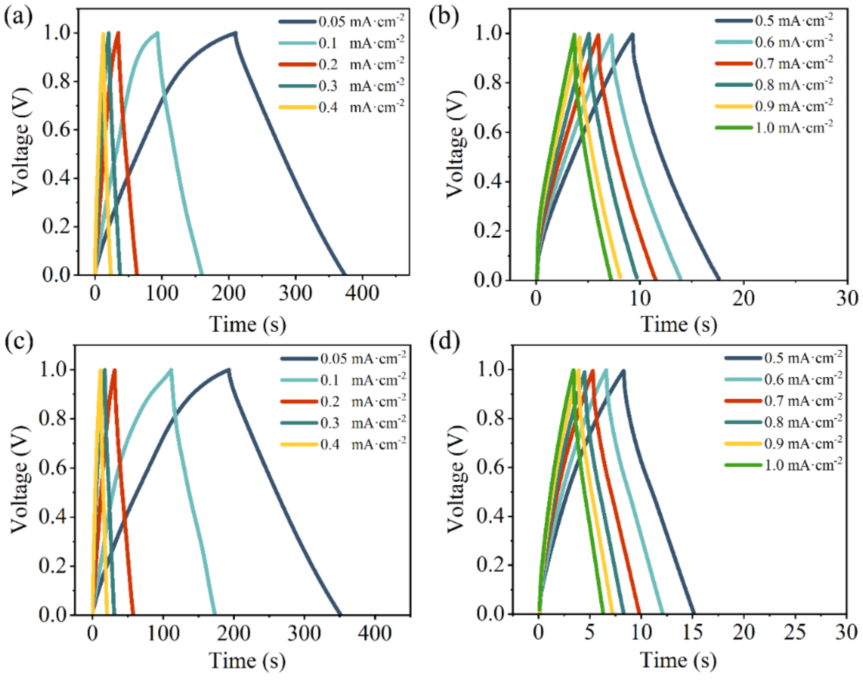

Figure S11. GCD curves of MSC_{500} at different current densities of (a) 0.05-0.4 $mA \cdot cm^{-2}$ and (b) 0.5-1.0 $mA \cdot cm^{-2}$. GCD curves of MSC_{1000} at different current densities of (c) 0.05-0.4 $mA \cdot cm^{-2}$ and (d) 0.5-1.0 $mA \cdot cm^{-2}$.

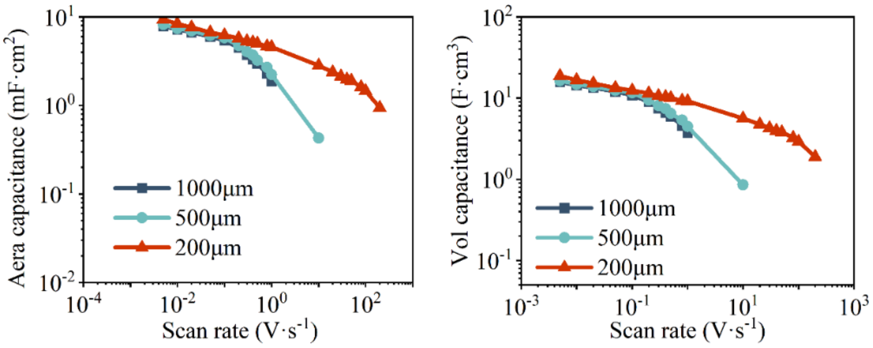

Figure S12. (a) Areal capacitance and (b) volumetric capacitance of MSCs based on CV curves.

Fig.S12 shows the areal capacitance and volumetric capacitance of graphene-based MSC_{1000}, MSC_{500}, and MSC_{200}. At a low scan rate of 5 mV·s^{-1}, the areal capacitance and volumetric capacitance of MSC_{200} are calculated to be 9.37 mF·cm^{-2} and 18.74 F·cm^{-3}, respectively, which are higher than those of MSC_{500} (8.20 mF·cm^{-2} and 16.40 F·cm^{-3}) and MSC_{1000} (7.84 mF·cm^{-2} and 15.68 F·cm^{-3}). Additionally, the rate capability of MSC_{200} is superior to that of MSC_{500} and MSC_{1000}. When the scan rate is increased, the capacitance of MSC_{200} slowly decreases, maintaining an areal capacitance of 1.46 mF·cm^{-2} and a volumetric capacitance of 2.91 F·cm^{-3} at a scan rate of 100 V·s^{-1}. Even at an ultra-fast scan rate of 200 V·s^{-1}, MSC_{200} retains an areal capacitance of 0.94 mF·cm^{-2} and a volumetric capacitance of 1.89 F·cm^{-3}. In sharp contrast, areal and volumetric capacitances of MSC_{500} are 0.43 mF·cm^{-2} and 0.85 F·cm^{-3} at a scan rate of 10 V·s^{-1}, and those of MSC_{1000} are 0.39 mF·cm^{-2} and 0.78 F·cm^{-3} at a scan rate of 3 V·s^{-1}. Therefore, the reduction in interdigitated width and spacing not only increases the charge storage capacity but also reduces the resistance to ion transport in the electrolyte.

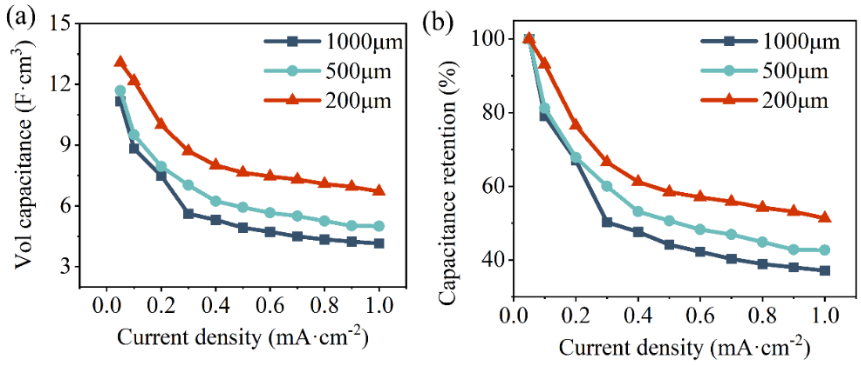

Figure S13. (a) Volumetric capacitance of MSC_{200} based on GCD curves. (b) Capacitance retention of MSC_{200} at different current densities relative to 0.05 mA·cm^{-2}.

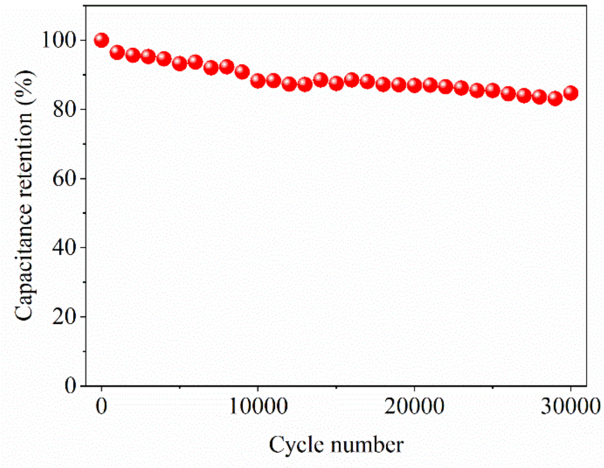

Figure S14. Cycling performance of MSC_{200} at the scan rate of 10 $V \cdot s^{-1}$.

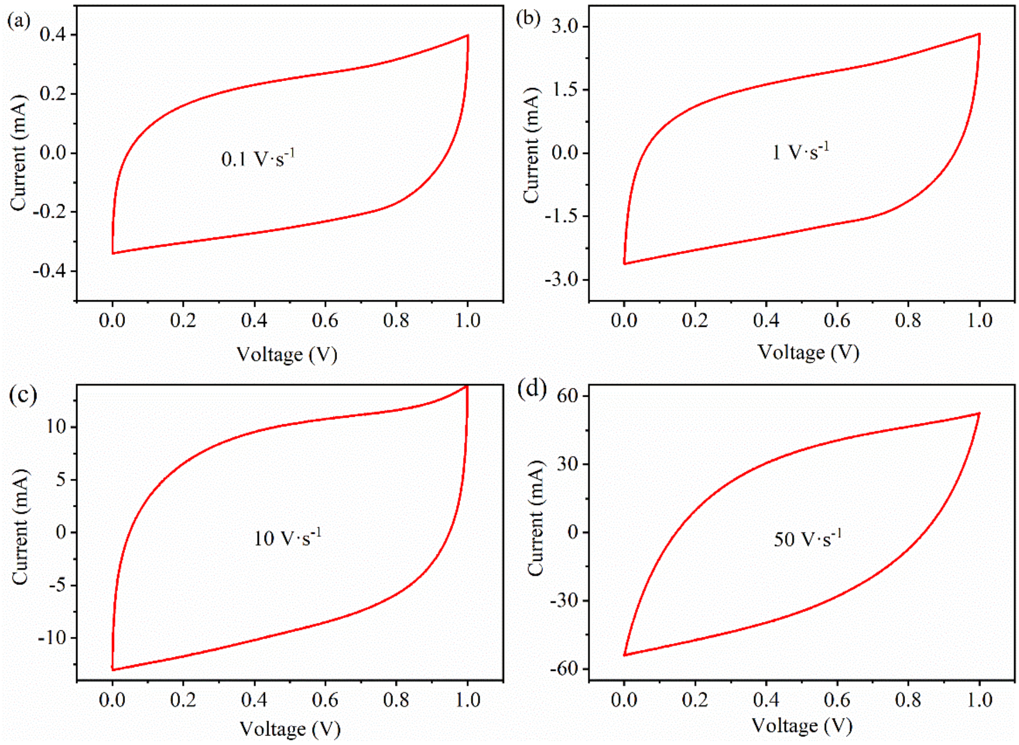

Figure S15. (a~ d) CV curves of MSC_{200} with a thickness of 7 μm at different scan rates of 0.1, 1, 10, 50 V·s-1.

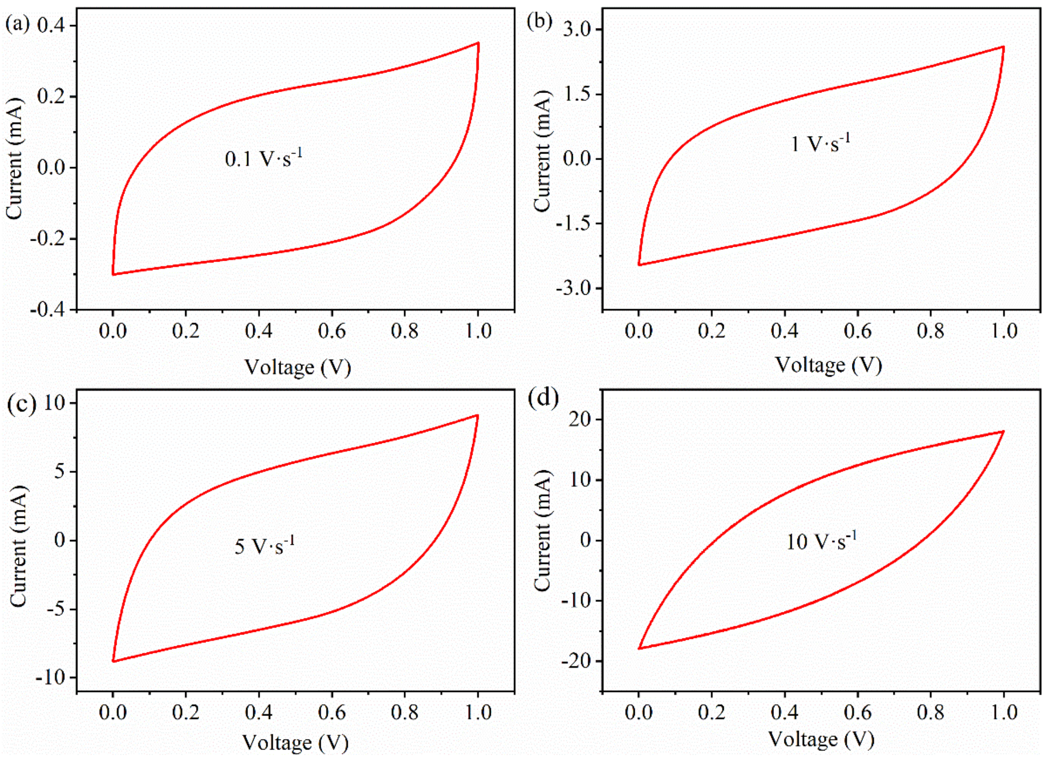

Figure S16. (a~ d) CV curves of MSC_{200} with a thickness of 10 μm at different scan rates of 0.1, 1, 5, 10 V·s-1.

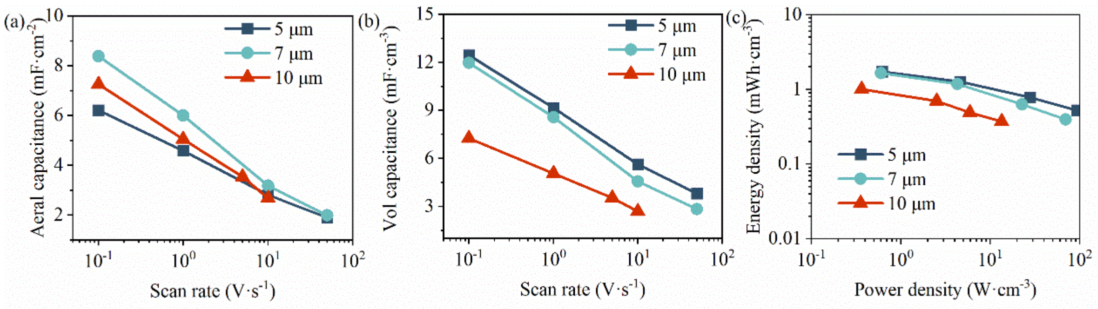

Figure S17. (a) Areal capacitance, (b) volumetric capacitance and (c) Ragone plot on energy density and power density of MSC_{200} with thicknesses of 5, 7, and 10 μm as a function of scan rate.

Table S3. Comparison of our work with the state-of-the-art MSCs based on carbon materials.

Electrode material	Electrolyte	Preparation method	Areal Capactitance ($mF\cdot cm^{-2}$)	Scan rate ($V\cdot s^{-1}$)	Energy density ($\mu Wh\cdot cm^{-2}$)	Power density ($mW\cdot cm^{-2}$)	Ref.
Graphene	PVA/H_2SO_4	Screen printing	1.36	0.9	/	/	[2]
Graphene/SWCNTS	PVA/H_3PO_4	Screen printing	1.324	20	0.361	20.13	[9]
Graphite/Carbon black	PVA/H_2SO_4	Screen printing	10.64	0.1	1.47	0.2	[10]
Graphene	EMM/TFSI/PS-PMMA-PS	Inkjet printing	0.268	1	0.035	0.32	[11]
Graphene	PVA/H_2SO_4	3D printing	56.7	0.2	7.8	31.7	[12]
Laser-scribed graphene	PVA/H_2SO_4	Laser-scribed	2.32	10	1.52	152	[13]
rGO	PVA/H_2SO_4	Laser pulses	6.3	1	0.6	0.6	[14]
CNTs	PVA/H_3PO_4	3D printing	4.69	0.1	/	/	[15]
Electrochemically exfoliated graphene	PVA/H_2SO_4	Mask spraying	7.6	0.5	/	/	[16]
Onion-like carbon	Et_4NBF_4/propylene carbonate	Electrophoretic deposition	1.7	200	1.0	110	[17]
graphene/CNTs	PVA/H_3PO_4	Screen printing	7.7	0.2	1.0	0.5	[18]
Graphene/Carbon black	PVA/H_3PO_4	Screen printing	1.0	0.5	0.09	0.12	[19]
3DGNs /SWNT/AgNW	PVA/LiCl	Plotter cutting	19	5	2.75	0.361	[20]
Graphene/Carbon black	PVA/H_2SO_4	Screen printing	9.15	200	1.3	89.9	This work

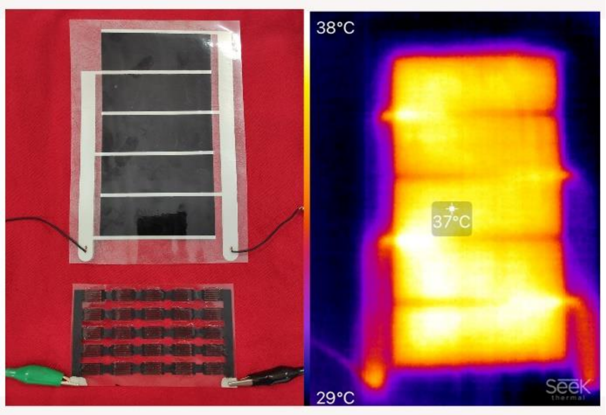

Figure S18. The modular MSCs power a wearable heater for uniform heating.

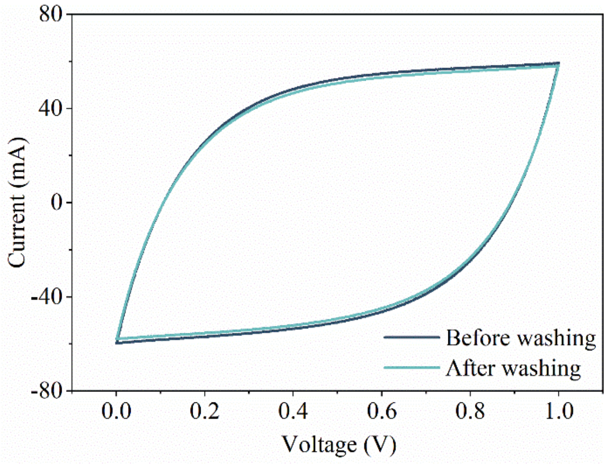

Figure S19. CV curves of PU-encapsulated MSC_{200} at $100V\cdot s^{-1}$ scan rate before and after washing.

Reference

1 E. B. Secor, S. Lim, H. Zhang, C. D. Frisbie, L. F. Francis and M. C. Hersam, *Adv. Mater.*, 2014, **26**, 4533–4538.

2 H. Chen, Y. Zhang, Y. Ma, S. Chen, Y. Wu, Y. Lu, H. Ren, S. Xin and Y. Bai, *Adv. Mater. Interfaces*, 2021, **8**, 2000888.

3 P. G. Karagiannidis, S. A. Hodge, L. Lombardi, F. Tomarchio, N. Decorde, S. Milana, I. Goykhman, Y. Su, S. V. Mesite, D. N. Johnstone, R. K. Leary, P. A. Midgley, N. M. Pugno, F. Torrisi and A. C. Ferrari, *ACS Nano*, 2017, **11**, 2742–2755.

4 K. Pan, Y. Fan, T. Leng, J. Li, Z. Xin, J. Zhang, L. Hao, J. Gallop, K. S. Novoselov and Z. Hu, *Nat. Commun.*, 2018, **9**, 5197.

5 K. Arapov, K. Jaakkola, V. Ermolov, G. Bex, E. Rubingh, S. Haque, H. Sandberg, R. Abbel, G. De With and H. Friedrich, *Phys. Status Solidi RRL*, 2016, **10**, 812–818.

6 L. Liu, Z. Shen, X. Zhang and H. Ma, *J. Colloid Interface Sci.*, 2021, **582**, 12–21.

7 D. S. Kim, J.-M. Jeong, H. J. Park, Y. K. Kim, K. G. Lee and B. G. Choi, *Nano-Micro Lett.*, 2021, **13**, 87.

8 W. J. Hyun, E. B. Secor, M. C. Hersam, C. D. Frisbie and L. F. Francis, *Adv. Mater.*, 2015, **27**, 109–115.

9 S. Bellani, E. Petroni, A. E. Del Rio Castillo, N. Curreli, B. Martín-García, R. Oropesa-Nuñez, M. Prato and F. Bonaccorso, *Advanced Functional Materials*, 2019, **29**, 1807659.

10 M. Wang, J. Wang, A. Wei, X. Li, W. Zhang and Y. Liu, *J. Alloys Compd.*, 2024, **976**, 173125.

11 W. J. Hyun, E. B. Secor, C. Kim, M. C. Hersam, L. F. Francis and C. D. Frisbie, *Adv. Energy Mater.*, 2017, **7**, 1700285.

12 W. Li, Y. Li, M. Su, B. An, J. Liu, D. Su, L. Li, F. Li and Y. Song, *J. Mater. Chem.A*, 2017, **5**, 16281–16288.

13 M. F. El-Kady and R. B. Kaner, *Nat. Commun.*, 2013, **4**, 1475.

14 D. Shen, G. Zou, L. Liu, W. Zhao, A. Wu, W. W. Duley and Y. N. Zhou, *ACS Appl. Mater. Interfaces*, 2018, **10**, 5404–5412.

15 W. Yu, H. Zhou, B. Q. Li and S. Ding, *ACS Appl. Mater. Interfaces*, 2017, **9**, 4597–4604.

16 X. Shi, Z. Wu, J. Qin, S. Zheng, S. Wang, F. Zhou, C. Sun and X. Bao, *Adv. Mater.*, 2017, **29**, 1703034.

17 D. Pech, M. Brunet, H. Durou, P. Huang, V. Mochalin, Y. Gogotsi, P.-L. Taberna and P. Simon, *Nat. Nanotechnol.*, 2010, **5**, 651–654.

18 J.-K. Chih, A. Jamaluddin, F. Chen, J.-K. Chang and C.-Y. Su, *J. Mater. Chem.A*, 2019, **7**, 12779–12789.

19 X. Shi, S. Pei, F. Zhou, W. Ren, H.-M. Cheng, Z.-S. Wu and X. Bao, *Energy Environ. Sci.*, 2019, **12**, 1534–1541.

20 S.-W. Kim, K.-N. Kang, J.-W. Min and J.-H. Jang, *Nano Energy*, 2018, **50**, 410–416.
